# Characteristics of Anterior Lens Opacities in Children

**DOI:** 10.2174/1874364101711010084

**Published:** 2017-04-28

**Authors:** Lena Dixit, Michael Puente, Kimberly G. Yen

**Affiliations:** 1 Department of Ophthalmology, Baylor College of Medicine, Houston, TX, USA; 2 Department of Ophthalmology and Pediatrics, Texas Children’s Hospital and Baylor College of Medicine, Houston, TX, USA

**Keywords:** Anterior polar cataract, Pediatric cataract, pediatric cataract surgery, Amblyopia, Cataracts, Anterior lens opacities

## Abstract

**Background:**

Anterior lens opacities (ALO) are found in 3-14% of pediatric patients with cataracts. No clear guidelines exist in the management and treatment of these cataracts.

**Objective:**

To evaluate pediatric patients with anterior lens opacities and assess rate of amblyopia and need for surgery over time.

**Methods:**

A retrospective chart review was performed on patients with unilateral and bilateral anterior lens opacities (ALOs) seen between January 2008 and December 2014. Size, location, and type of ALO were noted. Refractive error, necessity for treatment of amblyopia, and interventions were recorded.

**Results:**

A total of 31 patients were included in the study. 17 patients had unilateral ALOs and 14 had bilateral ALOs. The majority of the cataracts (90.3%) were centrally located. The most common type of cataract was the polar type of cataract and the vast majority (48.4%) was < 1mm in size. 38.7% of patients had concurrent ocular conditions and 9.7% had systemic associations. 28.6% of patients with bilateral cataracts and 35.3% of the patients with unilateral cataracts were treated for amblyopia. Three patients required cataract surgery.

**Conclusion:**

About half of anterior lens opacities are less than 1mm in size and the majority are of the polar type. Risk of amblyopia in these patients is higher than in the general population. Anisometropia is the most common cause of amblyopia. Ocular associations are seen at a relatively high frequency and systemic associations can occur but are uncommon. The need for surgical intervention is infrequent; however, growth of ALOs and associated cortical changes may be risk factors for surgery.

## INTRODUCTION

Congenital anterior lens opacities (ALOs) are characterized by a white opacity involving or just below the anterior lens capsule and are estimated to represent between up to 14% of all congenital cataracts [1]. Generally considered non-progressive, they are often bilateral, rarely larger than 3 mm in diameter, and have historically been considered visually insignificant [1, 2] However, various studies have found that over a quarter of patients with ALOs may develop amblyopia or strabismus, usually attributed to anisometropia [1, 3, 4]. Despite the high rate of amblyopia in these patients, no clear guidelines exist regarding which types of these patients are at risk for developing amblyopia. Because early detection is important in the management of amblyopia, awareness of the association with amblyopia in patients with ALOs is critical.

Three primary sub-types of congenital anterior cataracts are recognized: polar, subcapsular, and pyramidal. Polar cataracts are those that are round and central with a well-defined border and are the most common form of congenital anterior cataract. Cataracts that are not circular with defined margins are classified as subcapsular [4]. The pyramidal subtype is characterized by a conical projection into the anterior chamber arising from the anterior capsule of the lens and has been associated with a greater than 90% risk of amblyopia [3].

This study aims to evaluate the characteristics of and the interventions required in patients with congenital ALOs and aims to increase awareness of the need for early refraction and monitoring of amblyopia in these patients.

## METHODS

We performed a retrospective chart review of all patients who were seen at the ophthalmology clinic at Texas Children’s Hospital from January, 2008 to December, 2014 with congenital anterior lens opacities. Prior to initiating this study, approval from the Institutional Review Board of the Baylor College of Medicine was obtained to perform this study. The patients were identified through an ICD-9 search of the electronic medical records. Cataracts were classified according to their sub-type (polar, subcapsular, and pyramidal), size (<1 mm, 1-3 mm, or >3 mm), laterality, and location on the lens (central or para-central) based on previous classification in the literature [5]. Data were also collected regarding gender, age at diagnosis, length of follow-up, associated ocular or systemic conditions, ocular alignment, refractive error, presence and treatment of amblyopia, and surgical intervention. We defined anisometropia as ≥1.00 D of either sphere or cylinder.

## RESULTS

A total of 31 patients with anterior lens opacities were identified and included in the study. Mean age at presentation was 70.2 weeks old (range 2.0 – 344.6 weeks, SD 103.8 weeks). Twenty-two patients were female and 9 were male. A total of 14 patients had bilateral cataracts and 17 had unilateral cataracts. The average length of follow-up was 130.9 weeks (range 0 – 497.9 weeks, SD 114.8 weeks). The majority (28/31 or 90.3%) of patients had a centrally located cataract, while only 3 patients (9.7%) in the study had a para-central cataract. In the patients with bilateral cataracts, all patients had symmetric types and locations of their cataracts although the size of the cataracts was not always the same.

The polar type of cataract was the most common type in both the bilateral and unilateral groups. In the bilateral group, 85.7% (12/14) patients had polar type of cataracts, 14.3% (2/14) patients had pyramidal cataracts, and no patients had subcapsular cataracts.

In the patients with bilateral ALOs, 28.6% (4/14) developed anisometropic amblyopia. Characteristics of these patients are listed in Table (**1**). Two patients ultimately required surgery after failing medical management with glasses for anisometropia and subsequent patching.

In the patients with unilateral ALOs, 75.5% (13/17) patients had polar cataracts, 11.8% (2/31) patients had pyramidal cataracts, and 11.8% (2/17) patients had subcapsular cataracts. In this group, 35.3% (6/17) patients were treated for amblyopia. Table **2** lists the characteristics of these patients. One patient with deprivational amblyopia required cataract surgery.

The majority of the cataracts in both the unilateral and bilateral groups were <1mm in size. Patients who developed anisometropia in the bilateral group had in all size categories. The one patient with a cataract > 3 mm required surgery. All patients who developed anisometropia or amblyopia in the unilateral group had a cataract between 1-3 mm in size.

The highest amount of astigmatism measured in the bilateral ALO group was a patient with 4.00 diopters of cylinder in the right eye and 4.50 diopters in the left eye. The highest amount of astigmatism measured in the unilateral group was a patient with 4.00 diopters of cylinder in the ALO eye, and no astigmatism in the fellow, non-ALO eye.

The highest amount of hyperopia measured in the bilateral ALO group was a patient with +5.00 spherical diopters in the right eye, and +5.50 spherical diopters in the left eye. The highest amount of hyperopia measured in the unilateral group was +8.50 spherical diopters in the right eye (non-ALO eye), and +9.50 spherical diopters in the left eye (ALO eye).

The largest amount of anisometropia measured in the bilateral ALO group was a difference of 3.50 diopters of cylinder. The patient had a cylinder of 4.50 diopters in the right eye containing a >3 mm ALO, and a cylinder of 1.00 diopter in the left eye containing an ALO of 1-3 mm. The largest amount of anisometropia measured in the unilateral group was a difference of 4.00 diopters of cylinder. The patient had 4.00 diopters of astigmatism in the right eye containing an ALO of 1-3 mm, and no astigmatism in the left eye containing no ALO. This same patient also had the highest magnitude of astigmatism recorded in the group.

Strabismus was noted to occur in 9.7% (3/31) of our patients. Two patients with bilateral cataracts had strabismus, one with intermittent esotropia and a second with intermittent exotropia. One patient with a unilateral ALO had an intermittent esotropia. None of the patients required surgical intervention for the strabismus although both of the bilateral patients were treated for anisometropic amblyopia.

A total of 38.7% (12/31) of the patients had associated ocular findings, and 9.7% (3/31) had systemic associations. The ocular associations included adherent iris tissue, associated cortical or nuclear lens opacities, retinopathy of prematurity, strabismus, Peters anomaly, and Axenfeld-Reiger anomaly. The systemic associations included Down syndrome, tuberous sclerosis, and imperforate anus with middle ear anomalies (Table **3**).

A total of 3 patients required cataract surgery in our study. The patients that required surgical intervention in our study had either a pyramidal type of cataract or cortical changes associated with a polar type of cataract.

## DISCUSSION

Congenital anterior lens opacities are developmental anterior lens opacities that are felt to occur from mesodermal tissue that is trapped in the lens capsule during embryological development [4]. Traditionally, it has been felt that these cataracts have little to no visual significance [1, 3]. The cataracts are usually small anterior axial opacities which may be unilateral or bilateral [1, 3]. Generally, they remain stationary in size but may progress to requiring surgery [3]. Although need for cataract surgery is infrequent in these patients, progression or increase in the size of the cataracts has been described [3, 5].

The polar type of anterior lens opacity was the most common type of ALO in our study and this is consistent with previous reports [4]. Subscapsular cataracts were uncommon in our population with only two patients in our study having this subtype, although they have been reported to occur with higher frequency in other studies [4]. In the bilateral patients in this study, location of the cataract was similar in both eyes. About half of the patients had ALOs <1mm in size with very few having ALOs >3mm.

In our study, about a third of the patients required treatment for amblyopia, mainly due to anisometropia. Previous studies have also demonstrated a high incidence of refractive amblyopia in patients with ALOs [1, 3]. Incidence of amblyopia in a 2005 study by Ceyhan *et al.* was similar to ours and noted to be 28.8%;[4] similarly, Jaafar and Robb reported an overall incidence of amblyopia of 30.2% [1].

The reason for the development of anisometropia in these patients has not been clearly delineated; however an association of anterior lens opacities with corneal astigmatism has been described [6]. Anterior lens opacities have also been associated with keratoconus, corneal opacities, and other congenital anomalies, which is consistent with this theory [7]. Due to the changes on the lens, it is also possible that these patients have a component of lenticular astigmatism as well. The etiology for the higher amount of hyperopia in some of these patients is unclear.

Strabismus has been described to occur in patients with anterior lens opacities, although the rate of strabismus varies. While Jaafar and Robb found in their 1984 study that 11 of 63 patients with congenital anterior lens opacities developed strabismus, a separate review by Ceyhan *et al*. in 2005 showed that not one of their 59 patients with ALOs had developed strabismus [1, 4]. In our series, 9.7% (3/31) of the patients developed strabismus although none required surgical intervention for the strabismus.

Congenital ALOs have been associated with a wide variety of other ocular pathologies, including Peter’s anomaly, microcornea, persistent papillary membrane, aniridia, retinitis pigmentosa, retinoblastoma, and cornea guttata. [1, 5, 8, 9]. In our population, 38.7% of the patients had other ocular abnormalities in addition to the anterior lens opacities. Systemic associations with ALOs, while reported, are uncommon. None of the systemic associations in our study were life-threatening, although the association of ALO with retinoblastoma has been described in 2 separate studies [1, 4, 9].

Our study is limited by it small sample size, limited follow up, and retrospective nature. Due to the age of the patients, visual acuity was often not able to be measured beyond fix and follow and was therefore not evaluated in this paper. Because the patients were seen by a number of different providers, some data was limited as the medical record documentation was not standardized between providers. Finally, our patients did not have corneal topography performed, so differentiation of lenticular from corneal astigmatism could not be made.

Although anterior lens opacities are generally small, these lesions are associated with a high rate of amblyopia. The need for surgical intervention for the ALOs is infrequent, however some patients may progress to require surgery; pyramidal subtype or cortical changes may increase the risk of eventually needing surgery. The most common etiology of amblyopia in patients with ALOs is anisometropia. Therefore, patients with ALOs need to be monitored on a regular basis for development of anisometropia and amblyopia. Ocular associations are seen relatively frequently and systemic associations may exist but are uncommon.

## Figures and Tables

**Table 1:** Bilateral anterior lens opacities.

**Amblyopic patients**	**Number (percent)**
Anisometropic amblyopia	4 (28.6%)
Anisometropic hyperopia	1 (7.1%)
Anisometropic hyperopia + astigmatism	1 (7.1%)
Anisometropic myopia	1 (7.1%)
**Subtype in amblyopic patients**	
Polar type	3 (21.4%)
Pyramidal type	1 (7.1%)
**Surgical intervention required**	2 (14.,3%)
Pyramidal	1
Polar	1
**Size of cataract**	
<1mm	8 (57.1%)
1-3mm	5 (35.7%)
>3mm	1 (7.1%)

**Table 2:** Unilateral anterior lens opacities.

**Amblyopic patients**	**Number (percent)**
Deprivational amblyopia	1 (5.9%)
Anisometropic amblyopia	5 (29.4%)
Anisometropic hyperopia	2 (11.8%)
Anisometropic hyperopia + astigmatism	2 (11.8%)
**Subtype in amblyopic patients**	
Polar type	2 (11.8%)
Pyramidal type	1 (5.9%)
Pyramidal + subcapsular	1 (5.9%)
**Surgical intervention required**	1 (5.9%)
Polar	1 (5.9%)
**Size of cataract**	
<1mm	7 (41.2%)
1-3mm	10 (58.8%)
>3mm	0

**Table 3:** Ocular and Systemic Associations with Anterior Lens Opacities.

Ocular Associations	Number of patients *
Adherent iris tissue	3
Cortical cataract	2
Nuclear cataract	2
Retinopathy of prematurity	2
Strabismus	3
Peters Anomaly	1
Rieger’s Anomaly	1
Systemic Associations	Number of patients*
Downs Syndrome	1
Tuberous Sclerosis	1
Imperforate Anus	1
Middle ear abnormalities	1

*some patients had more than one association.
